# Antibacterial and wound healing potential of biosynthesized zinc oxide nanoparticles against carbapenem-resistant *Acinetobacter baumannii*: an in vitro and in vivo study

**DOI:** 10.1186/s12934-024-02538-3

**Published:** 2024-10-16

**Authors:** Mohamed I. Selim, Fatma I. Sonbol, Tarek E. El‑Banna, Walaa A. Negm, Engy Elekhnawy

**Affiliations:** 1 Pharmaceutical Microbiology Department, Faculty of Pharmacy, Tanta University, Tanta, 31527 Egypt; 2 Department of Pharmacognosy, Faculty of Pharmacy, Tanta University, Tanta, 31527 Egypt

**Keywords:** Nanoparticles, Carbapenem, Algae, *Spirulina*, LC/MS, Burn model

## Abstract

Carbapenem-resistant *Acinetobacter baumannii* denotes a significant menace to public health, and it mandates an urgent development of new effective medications. Here, we aimed to estimate the efficiency of the zinc oxide nanoparticles (ZnO NP) biosynthesized from *Arthrospira maxima* (*Spirulina*) both in vitro and in vivo. Carbapenem-resistant *A. baumannii* isolates were collected, identified, tested for their antibiotic susceptibility, and then subjected to PCR to detect carbapenemase-producing genes. The most predominant carbapenemase resistance gene was *bla*_KPC_. The biosynthesized ZnO NP were characterized using UV, FTIR, XRD, SEM, and TEM. The prepared ZnO NP was then tested against *A. baumannii* isolates to determine the minimum inhibitory concentration (MIC), which ranged from 250 to 1000 μg/ml. Burn wound was persuaded in twenty rats and inoculated with carbapenem-resistant *A. baumannii* isolate. Rats were allocated into four groups: a negative control group, a positive control group treated with topical 0.9% saline, a test treatment group that received topical ZnO NP, and a standard treatment group. All groups received treatment for 15 consecutive days and then euthanized. Skin samples were harvested and then subjected to histopathological and immunochemical investigations. ZnO NP revealed a comparable antibacterial activity to colistin as it revealed a lower level of fibrosis, mature surface epithelization with keratinization, and restoration of the normal skin architecture. In addition, it significantly decreased the immunoreactivity of the studied inflammatory markers. Thus, ZnO NP synthesized by *A. maxima* could be considered a promising, safe, and biocompatible alternative to traditional antibiotics in the therapy of carbapenem-resistant *A. baumannii* infections.

**Supplementary Information:**

The online version contains supplementary material available at 10.1186/s12934-024-02538-3.

## Introduction

The growing rate of resistance to numerous antibiotics is a significant hazard to public health all over the world. Many researchers are trying to develop new approaches to combat this phenomenon [1]. *Acinetobacter baumannii* is best known for its high antibiotic resistance. *Acinetobacter baumannii* is a Gram-negative coccobacillus. It is a non-motile, aerobic, rod-shaped bacterium linked mostly with life-threatening infections, mainly in health settings. It has a high incidence in immunocompromised patients, especially those who have stayed in the hospital for a long time [2]. In the last years, it has been considered a great threat to health and a “red alert” pathogen owing to its multidrug resistance (MDR) to different classes of antibiotics [3]. About 2% of the nosocomial infections in the USA are attributed to* A. baumannii.* However, such percentage doubles in the Middle East and Asia [4, 5].

Carbapenems are a powerful option for treating multidrug-resistant Gram-negative bacteria like *A. baumannii* infections. However, the extensive use of carbapenems has resulted in repeated outbreaks with carbapenem-resistant *A. baumannii* (CRA) [6]. There are multiple mechanisms adopted by *A. baumannii* to become resistant to Carbapenems. Carbapenemase production, porin mutations, and efflux pumps are the main mechanisms and plasmid-mediated carbapenemase production is the primary mechanism by which the pathogen resists carbapenems [7]. There are many genes encoding carbapenemases like *bla*-*Klebsiella pneumonia* carbapenemase (*bla*_KPC_), *bla*-oxacillin hydrolyzing enzymes-48 (*bla*_OXA-48_), *bla*-New Delhi metallo-β-lactamase (*bla*_NDM_), *bla*-active on imipenem (*bla*_IMP_), and *bla*-Verona integron-mediated metallo-β-lactamase (*bla*_VIM_) [8].

Some novel non-antibiotic approaches are being investigated for their effectiveness as antimicrobials. One of these approaches is the use of green algae and nanoparticles. Using algal cells for the green synthesis of nanoparticles provides many benefits, such as high biomass production, cost-effectiveness, and environmental sustainability. Algae offer variable metabolites essential for the stabilization of nanoparticles. Algae can also be cultivated in various conditions, including in waste environments. The extraction process is simple and can produce biocompatible metallic oxide nanoparticles [9]. *Arthrospira maxima* (*Spirulina)* is a blue-green algae with high protein content, essential minerals, and vitamins [10]. It has also shown antibacterial activity and an immune-enhancing efficacy [11].

Many researchers are considering green zinc oxide nanoparticles (ZnO NP) for their activity on bacteria as they are safer and less toxic than chemically synthesized ones [12, 13]. There are many advantages to using zinc over other metallic oxide nanoparticles. They are generally more biocompatible and have lower toxicity than other metallic oxides, rendering them suitable for medical and biological applications. They are also chemically stable [14]. They also have strong antimicrobial properties and are effective against a wide range of bacteria and fungi, which is beneficial for applications in medical devices, coatings, and textiles [15]. Zinc is more abundant and less expensive than other metals, such as gold or platinum, making ZnO NP a cost-effective choice for large-scale applications. These characteristics make zinc oxide nanoparticles a versatile and economical option for various industrial, environmental, and biomedical applications.

ZnO NP exhibits different mechanisms of action that include the formation of reactive oxygen species as the pathway of bactericidal action, generating reactive oxygen species (ROS), and the release of zinc ions (Zn2 +), which cause cell membrane damage and interrupt the metabolic pathways [16]. However, ZnO NP can be toxic to aquatic organisms such as fish, algae, and invertebrates. They also can cause soil contamination, altering its properties and negatively affecting the microbial life essential for soil health and fertility. Furthermore, ZnO NP can react with other environmental chemicals, potentially forming harmful byproducts [17].

We aimed to evaluate the antibacterial efficacy of ZnO NP biosynthesized by *A. maxima* against CRA in vitro and an infected burn model in rats. Furthermore, we elucidated the phytochemical profile of this green microalgae.

## Materials and methods

### Isolation of CRA and determination of the antibiotic resistance profile

#### Collection and identification of CRA

Twenty CRA isolates were obtained from different clinical samples from Cairo University Hospitals. As exposed in Table S1, specimens were isolated from blood, wound, urine, and sputum, pus. *A. baumannii* isolates were assessed for resistance to carbapenems by antibiotic breakpoint testing and considered resistant if they had a minimum inhibitory concentration (MIC) ≥ 8 μg/ml [18]. Resistant isolates were cultured in nutrient broth (Himedia, India) and stored in glycerol stock (50% glycerol/nutrient broth) at – 80 °C for further investigations.

#### Antibiotic susceptibility

It was implemented using the Kirby-Bauer disk method on Muller-Hinton agar (Himedia, India). Confirmation of carbapenem resistance was performed using meropenem (MEM, 10 µg) and imipenem (IPM, 10 µg) discs. Fifteen antibiotics were employed and they were colistin (CL, 10 µg), gentamicin (CN, 120 µg), amikacin (AK, 30 µg), cefazolin (CZ, 30 µg), cefuroxime (CXM, 30 µg), cefotaxime (CTX, 30 µg), ceftriaxone (CRO, 30 µg), ceftazidime (CAZ, 30 µg), cefepime (FEP, 30 µg), ciprofloxacin (CIP, 5 µg), levofloxacin (LEV, 5 µg), doxycycline (DO, 30 µg), piperacillin/tazobactam (TPZ, 100/10 µg), ampicillin/sulbactam (SAM, 10/10 µg), and cotrimoxazole (SXT, 23.75/1.25 µg).

#### Polymerase chain reaction (PCR)

Table S2 shows the primer sequence for the tested carbapenem resistance genes [19]. After the PCR process was completed, the PCR products were exposed to electrophoresis. The PCR products, DNA ladder, and negative control were all loaded to 1.2% agarose gel, and the power supply was set to 80 V for one hour. After completion, the gel was inspected for results on a UV transilluminator, and positive results were indicated by the detection of single sharp bands with a definite amplicon size for each gene.

### Preparation and characterization of green ZnO NP

#### Preparation and characterization of *A. Maxima*

The blue-green algae *A. maxima* was obtained from Bozhou Swnf Commercial and Trade Co., Ltd., China. *A. maxima* identification was carried out by Dr. Esraa Ammar at the Faculty of Science and was assigned an ascension number 2022-01- PG- W-67. Cold maceration extraction was done using 100 g of powdered dry microalgae and methanol as a solvent-to-feed ratio of 10 ml/g for 72 h. The recovered extract (26.4 g) was stored for further phytochemical and biological studies.

LC–MS/MS exploration of *A. maxima* was performed by implementing methods stated before [20, 21]. The negative electrospray ionization approach was utilized to detect the various phytoconstituents of the *A. maxima* extract. Targeted constituents were determined by comparing LC/MS data with previously published substances and reference databases [22, 23]

#### Preparation of ZnO NP

As previously reported, ZnO NP was synthesized using a wet chemical method [24]. Briefly, 196 ml of filtered algal biomass was added dropwise to zinc acetate di-hydrate. Then, at 30 °C, the mix was stirred, and the subsequent precipitate was oven-dried at 100 °C overnight.

#### Characterization of ZnO nanoparticles

Several tests were carried out to prove the process of producing ZnO NPs. The ZnO NP was characterized using techniques such as SEM/TEM for morphology, XRD for crystallinity, EDX/FTIR for chemical composition, and UV–vis spectroscopy for characterizing nanoparticles' optical properties and electronic structure.

UV–Vis spectroscopy was performed at a wavelength of (200–800) nm using a DS5 UV–Vis spectrophotometer (Edinburgh Instruments, UK). Detailed insights into dispersion, aggregation, or flocculation were tested by determining the zeta potential using a zeta sizer (Malvern Panalytical, UK) [25].

An XRD was accomplished using a Diffractometer system (Malvern Panalytical, UK) to check the purity and nanostructural properties. The system used two thetas (20–80°), with a minimum step size of two Theta: 0.001, and at a wavelength (Kα) = 1.54614°.

The morphology of nanoparticles was assessed using a field emission scanning electron microscope (FESEM) (Thermo Scientific, USA). The elemental structure was also assessed using energy dispersive X-ray (EDX).

Transmission electron microscopy (TEM) was performed on a Talos F200i TEM (Thermo-fisher, USA) at an accelerating voltage of 200 kV to elucidate the nanoparticle's structural morphology.

The composition of ZnO NP was also investigated by Fourier Transform Infrared (FTIR) analysis (ALPHA II Compact FTIR Spectrometer, Bruker, USA) in a range of 4000–1000 cm^−1^.

### ZnO NP antibacterial action (in vitro*)*

This was accomplished against the 20 isolates of *A. baumannii* by broth microdilution method starting with a concentration of 2000 μg/ml in the first well and then subjected to two-fold serial dilution with well 11 considered as positive control (bacterial culture and nutrient broth) and well 12 as negative control (nutrient broth only) [26, 27]. The minimum inhibitory concentration (MIC) was identified as the least concentration of ZnO NP that inhibited the bacterial growth following plates’ incubation for 24 h at 37 °C [28, 29].

### ZnO NP antibacterial action (in vivo*)*

#### Murine burn model design

Twenty male albino rats (weighted 120–150 g, 6–8 weeks old) from Cairo, Egypt, were housed under standard conditions [30, 31]. Animals were tested following the principles of the Research Ethical Committee, Faculty of Pharmacy, Tanta University, Tanta, Egypt (TP/RE/5/24 p-01).

Animals were anesthetized, and hair was removed from a circular area of two centimeters on the back of the rat. The area was then cleaned with 70% ethyl alcohol. The burn was induced by heating a metal spatula and applying it to the area for 30 s. Infection was induced by inoculating the burned area with 10 μl of 1 × 10^8^ CFU/ml *A. baumannii* (susceptible to colistin). After 24 h, the area was reinoculated with another 10 μl of the bacterial culture [32].

Four groups (n = 5) of animals were tested. The first was the negative control, which wasn’t infected and received 0.9% saline topically. The second group was treated with 0.9% saline topically (positive control). The third group was treated with topical 0.19% colistin (standard treatment group) [33]. The last group was treated with 0.2% ZnO NP topically (test group) [34]. All groups were treated for 15 consecutive days. On the 16th day, all of them were sacrificed, and skin samples were incised for histopathological and immunohistochemical (IHC) studies.

Acute toxicity studies were performed before the experiment for selection of the utilized dose according to Organization of Economic Co-operation and Development guidelines for testing chemicals by applying 0.2% ZnO NP topically [35]. The animals were observed individually for signs of toxicity within the first 30 min after dosing. Periodically in the first 24 h, with a particular awareness during the first 4 h. After that, animals were observed daily for a total period of two weeks.

#### Histopathological and IHC staining

After sacrificing rats, excised skin samples were retained in formalin. After the previously reported steps, the skin sections were stained with hematoxylin and eosin (H&E) and Masson’s trichrome stain for collagen fibers detection [36]. IHC involves the detection of tumor necrosis factor-alpha (TNF-alpha) and interleukin-6 (IL-6) immune-reactive cells in the skin sections.

### Statistical analysis

Results were illustrated as mean ± standard deviations (SD). Data was evaluated through ANOVA by GraphPad Prism 9.

## Results

### Sensitivity to antibiotics

**Fig. 1:**
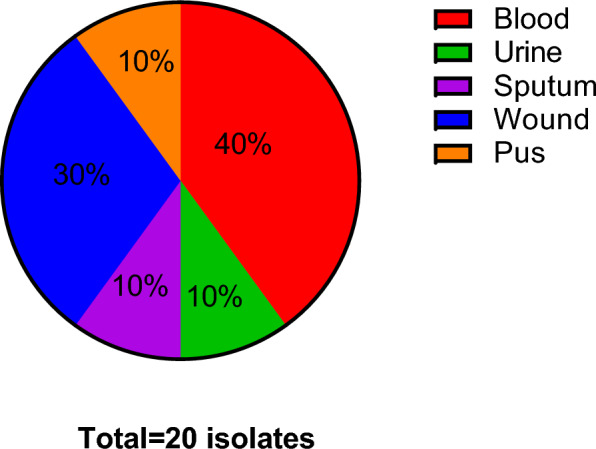
Pie chart showing the different sample types of tested isolates

CRA isolates from blood, wound, urine, sputum, and pus are shown in Fig. 1.

**Fig. 2:**
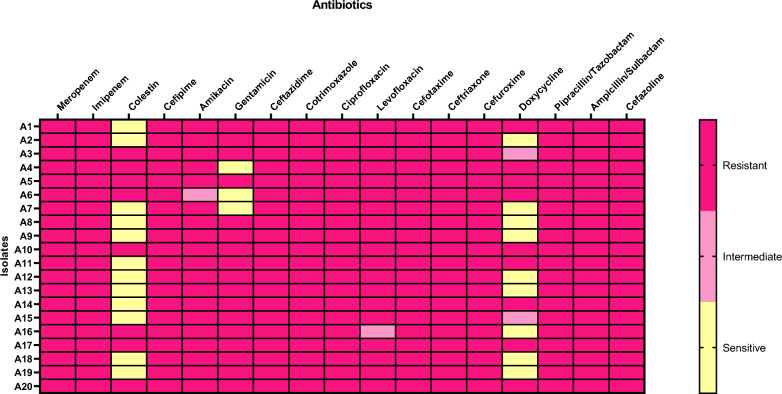
Heat map illustrating antibiotic susceptibility of the tested isolates

In addition to meropenem and imipenem, all isolates were resistant to ampicillin/sulbactam, piperacillin/tazobactam, sulfamethoxazole/trimethoprim, ciprofloxacin, cefazolin, cefuroxime, ceftriaxone, ceftazidime and cefepime. Eight (40%) and three isolates (15%) were resistant to colistin and gentamicin, respectively. However, 19 isolates (95%) were resistant to amikacin and levofloxacin. Nine isolates (45%) showed resistance toward doxycycline. Figure 2 illustrates the results of antibiotic susceptibility testing.

### PCR analysis

As exposed in Table S3, the most predominant carbapenemase resistance gene was *bla*_KPC_ as 17 (85%) isolates harbored it. The second prevailing gene was *bla*_VIM_, which was harbored in 13 (65%) isolates. In addition, *bla*_NDM-1_ was prevalent in 11 isolates (55%). The *bla*_OXA-48_ was detected in eight isolates (40%). The last detected gene was blaIMP, absent in nearly all isolates except for three (15%).

### Characterization of *A. maxima* and ZnO NP

Using LC–MS/MS (negative mode), 15 compounds were tentatively recognized in *A. maxima*. The main substances are amino acids such as 5-oxoproline, fatty acids such as gamma-linolenic acid, palmitic acid, and heptadecanoic acid, coumarins such as esculin, isoflavonoids such as daidzein-8-c-glucoside, flavonoids such as baicalein-7*-o-*glucuronide and kaempferol-3-*o-*α-l-rhamnoside, xanthine, and carboxylic acid such as succinic acid. The metabolite profile is illuminated in Table 1, whereas the negative mode total ion chromatograms (TIC) of *A. maxima* are displayed in Figure S1, representing the compounds detected.

**Table 1:** Metabolite profiling of *A. maxima* by LC–ESI–MS/MS analysis (negative mode ESI)

Peak No	Rt (min)	Precursor *m/z* [M-H]^−^	Error ppm	Compound Name	Molecular Formula	MS/MS	Ontology
1	1.09	117.01	− 0.2	Succinic acid	C_4_H_6_O_4_	73.21, 100.00, 117.07	Dicarboxylic acids and derivatives
2	1.19	128.03	− 0.6	5-Oxoproline	C_5_H_7_NO_3_	60.99, 68.99, 82.03, 84.99, 128.03	Alpha amino acids and derivatives
3	1.3	151.02	0.3	Xanthine	C_5_H_4_N_4_O_2_	65.99, 108.02	Xanthines
4	1.71	130.08	0.7	trans-4-Hydroxy-l-proline	C_5_H_9_NO_3_	132.09	Proline derivatives
5	2.01	146.04	0.7	d-Glutamate	C_5_H_9_NO_4_	54.03, 59.01, 71.01, 74.02, 100.04, 102.05, 116.04, 128.03	Glutamic acid and derivatives
6	4.43	339.07	0.4	Esculin	C_15_H_16_O_9_	69.01, 121.09, 178.84, 320.10, 339.34	Coumarin glycosides
7	12.31	445.07	− 3.7	Baicalein-7-*o*-glucuronide	C_21_H_18_O_11_	102.95, 191.05, 269.02, 377.07, 445.14	Flavonoid-7-*o*-glucuronides
8	15.18	431.12	− 0.7	Kaempferol-3-*o*-α-l-rhamnoside	C_21_H_20_O_10_	89.03, 285.10, 313.07, 395.01, 430.86, 431.11	Flavonoid-3-*o*-glycosides
9	17.71	415.18	0.6	Daidzein-8-c-glucoside	C_21_H_20_O_9_	267.13, 295.01, 325.12, 415.198	Isoflavonoid c-glycosides
10	18.96	277.21	0.3	Gamma-Linolenic acid	C_18_H_30_O_2_	59.01, 233.22	Lineolic acids and derivatives
11	19.92	253.05	1.2	Daidzein	C_15_H_10_O_4_	135.00, 184.93, 208.93, 225.04, 253.04	Isoflavones
12	21.30	279.23	0.5	Linoleic acid	C_18_H_32_O_2_	59.01, 261.22	Fatty Acids
13	13.88	283.05	5.1	Acacetin	C_16_H_12_O_5_	151.08, 252.13, 240.04, 268.03, 283.26	4′-o-methylated flavonoids
14	23.61	255.23	0.5	Palmitic acid	C_16_H_32_O_2_	237.22	Straight-chain fatty acid
15	26.08	269.24	0.7	Heptadecanoic acid	C_17_H_34_O_2_	269.24, 169.06, 94.93	Long-chain fatty acids


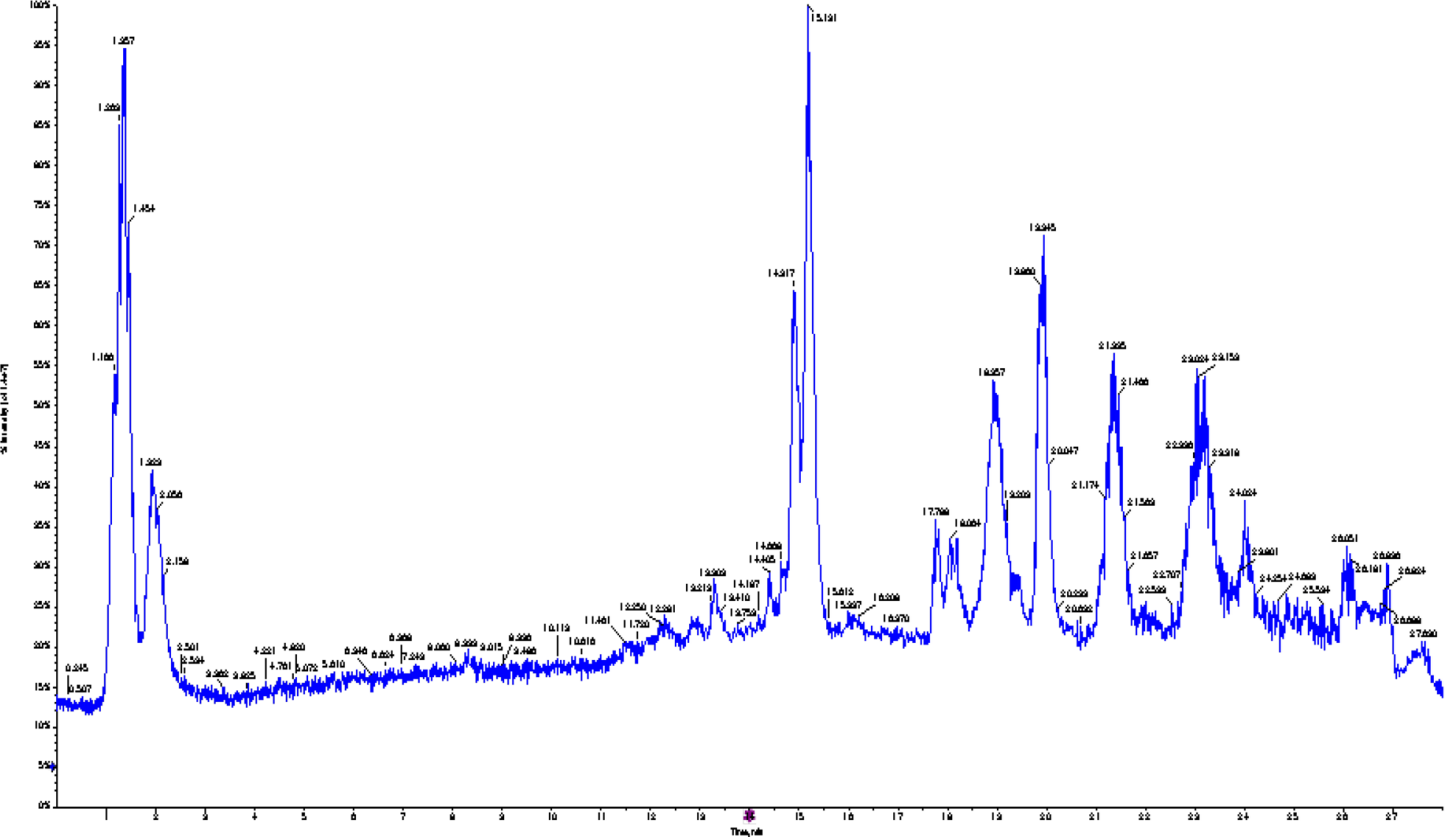


**Fig. 3:**
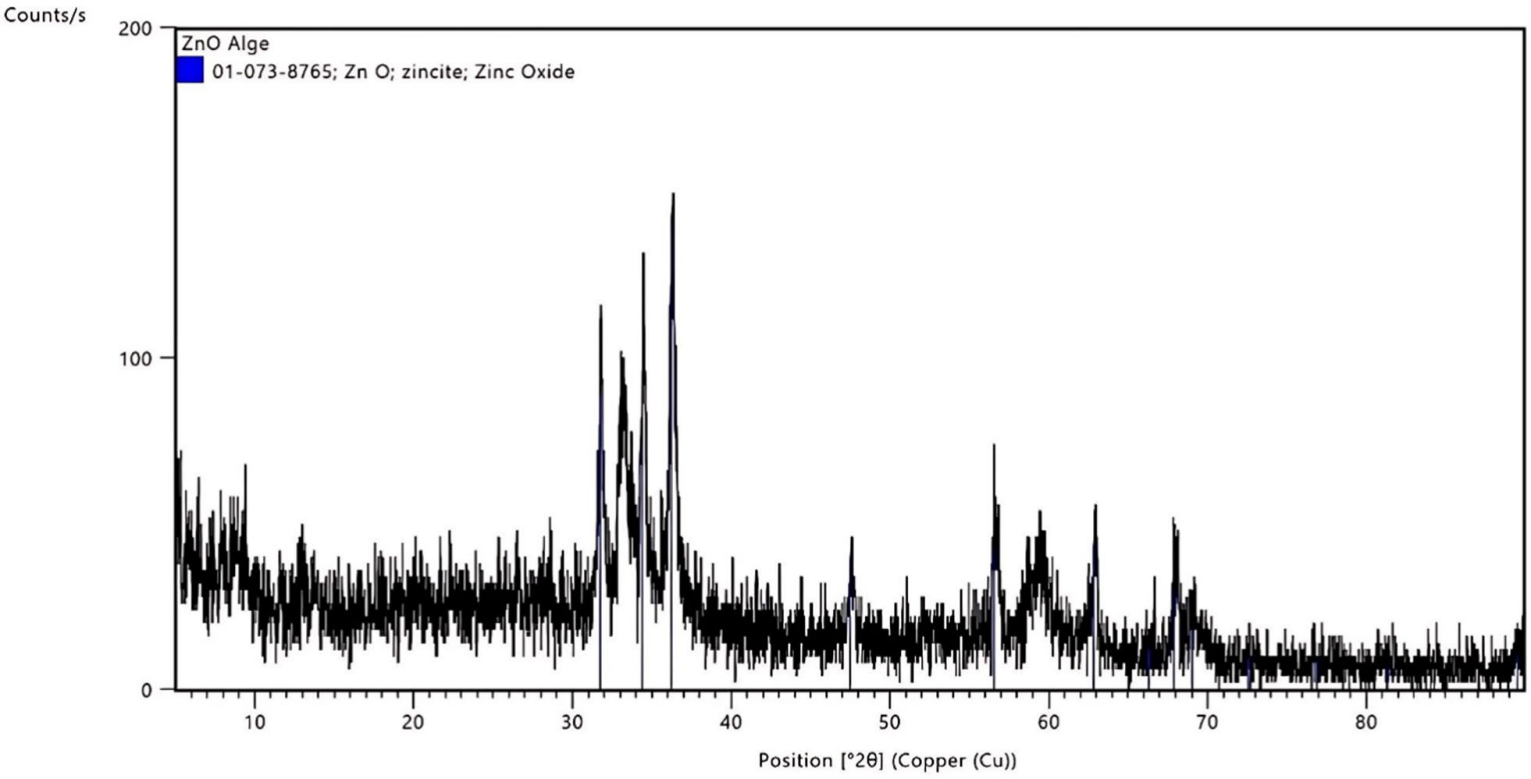
The XRD patterns of the biosynthesized ZnO NP

Figure 3 shows the XRD patterns of the ZnO NP. The XRD graph shows a pattern typical of a crystalline material, specifically ZnO. The graph shows several sharp peaks, indicating a crystalline material. These sharp peaks confirm that the sample is crystalline ZnO, most likely in the wurtzite structure, which is typical for ZnO. The matching of peaks with the reference pattern suggests that the sample predominantly comprises ZnO without significant impurities or secondary phases. Good alignment between the experimental and reference peaks indicates a high-quality ZnO sample with well-defined crystal planes [34].

**Fig. 4:**
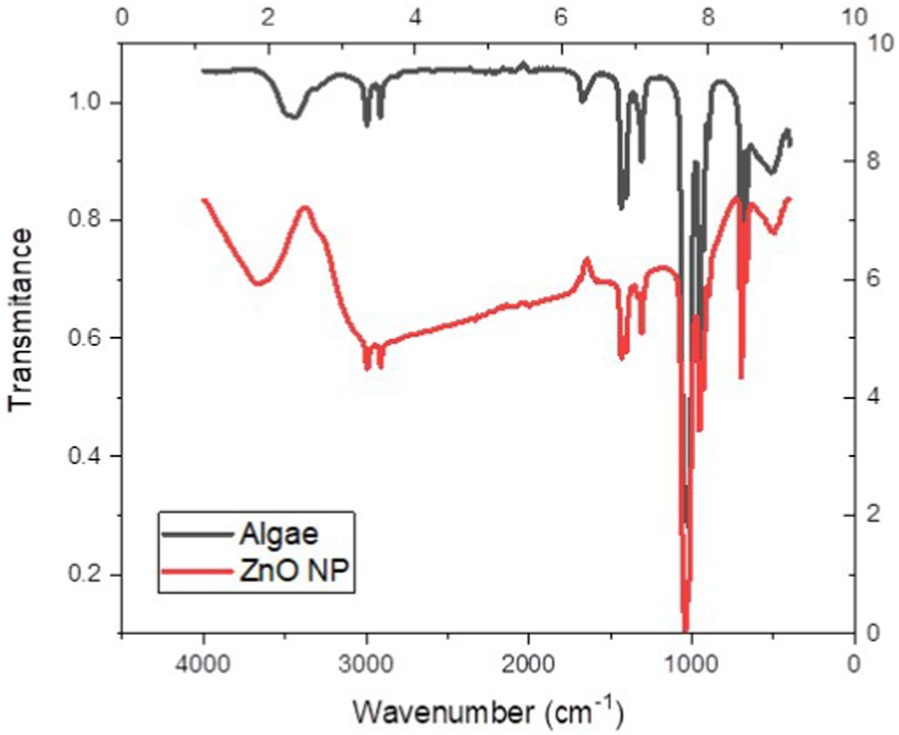
The FTIR spectrum of *A. maxima* and ZnO NP

The FTIR spectrum overlapped the algae and the ZnO NP, as shown in Fig. 4. The FTIR spectrum reveals distinct functional groups for “Algae” and “ZnO NP.” The algae spectrum is characterized by broad O–H stretching (~ 3300 cm^−1^), indicating hydroxyl groups or water, and C–H stretching (~ 2900–2850 cm^−1^), typical of aliphatic chains. Strong peaks at ~ 1650 cm^−1^ and ~ 1540 cm^−1^ suggest the presence of proteins (amide I and II bands), while C–H or O–H bending (~ 1400 cm^−1^) and C–O–C stretching (~ 1100–1000 cm^−1^) point to carboxylic acids, phenolics, and polysaccharides. In contrast, the ZnO NP spectrum shows O–H stretching (~ 3400 cm^−1^), likely from surface hydroxyl groups, and distinct Zn–O stretching vibrations (~ 1000–500 cm^−1^), characteristic of ZnO NP [34]. Overall, the algae spectrum shows typical biological signatures, while the ZnO NP spectrum is dominated by the Zn–O bonds specific to zinc oxide.

The UV spectrum displayed an absorption peak at 362 nm, and zeta potential results were − 5.70 ± 1.3559, which indicates a fair repulsion and low aggregation.

**Fig. 5:**
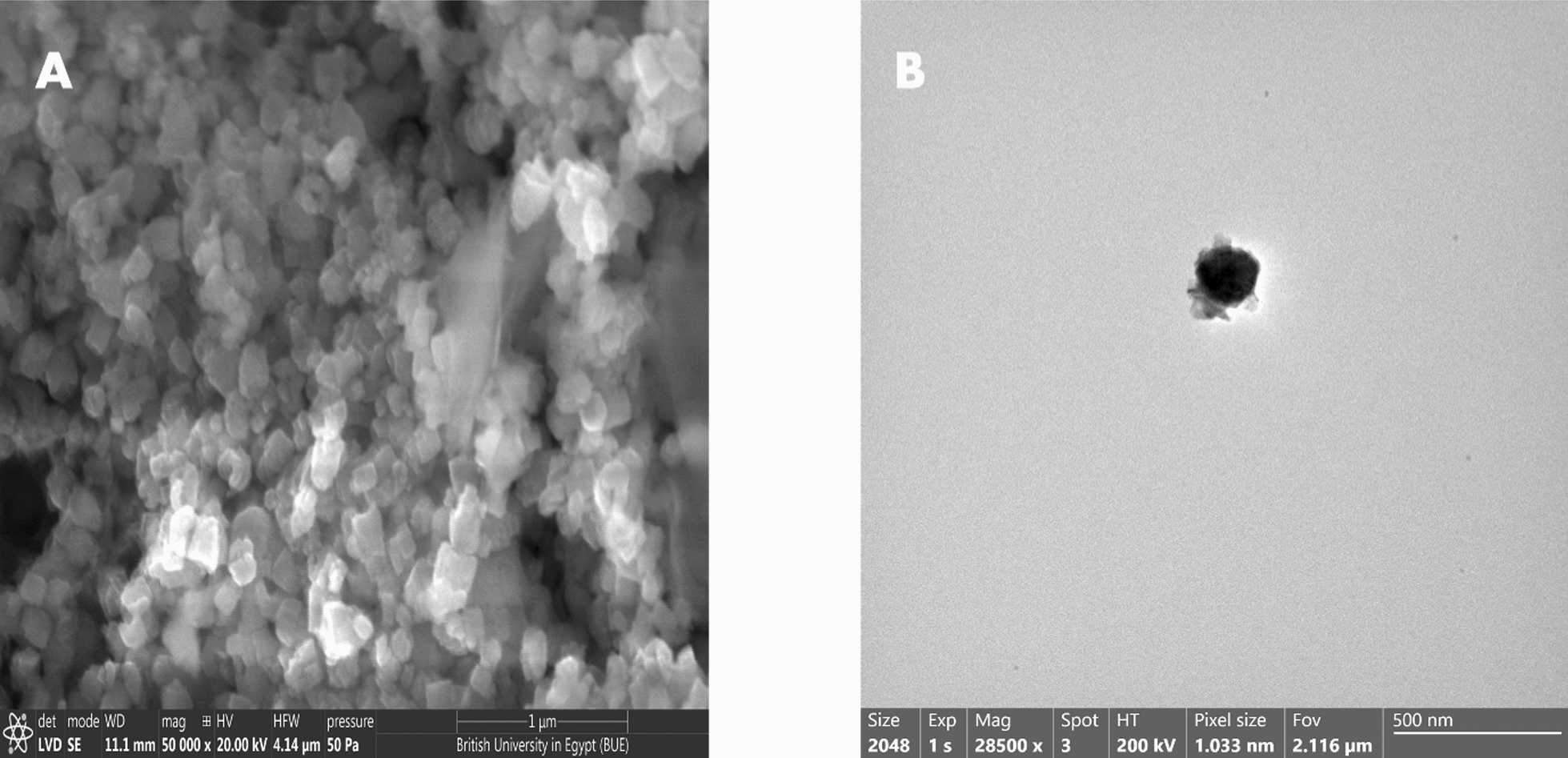
SEM (**A**) and TEM (**B**) micrographs of the ZnO NP

The SEM image exhibited that ZnO NP had a good dispersion and was spherical with quite aggregations (Fig. 5A). The TEM image of the ZnO NP (Fig. 5B) revealed that the prepared particles were in the nanoscale with a size less than 100 nm.

### Antibacterial activity of ZnO NP

The MIC values of the ZnO NPs ranged from 250 to 1000 μg/ml (Table 2).

**Table 2:** The MIC values of ZnO NP toward *A. baumannii* isolates

Isolate code	MIC for ZnO NP (µg/ml)	Isolate code	MIC for ZnO NP (µg/ml)
**A1**	500	**A11**	250
**A2**	1000	**A12**	500
**A3**	250	**A13**	500
**A4**	500	**A14**	500
**A5**	500	**A15**	500
**A6**	1000	**A16**	500
**A7**	500	**A17**	250
**A8**	500	**A18**	500
**A9**	500	**A19**	500
**A10**	500	**A20**	500

Most of the isolates (15 out of 20) (75%) required 500 µg/ml of ZnO NP to inhibit their growth, indicating that this concentration is effective against a wide range of bacterial strains in this study. Only three isolates (15%) were inhibited at a lower concentration (250 µg/ml), while two isolates (10%) required a higher concentration (1000 µg/ml) for inhibition.

### Infected burn model

**Fig. 6:**
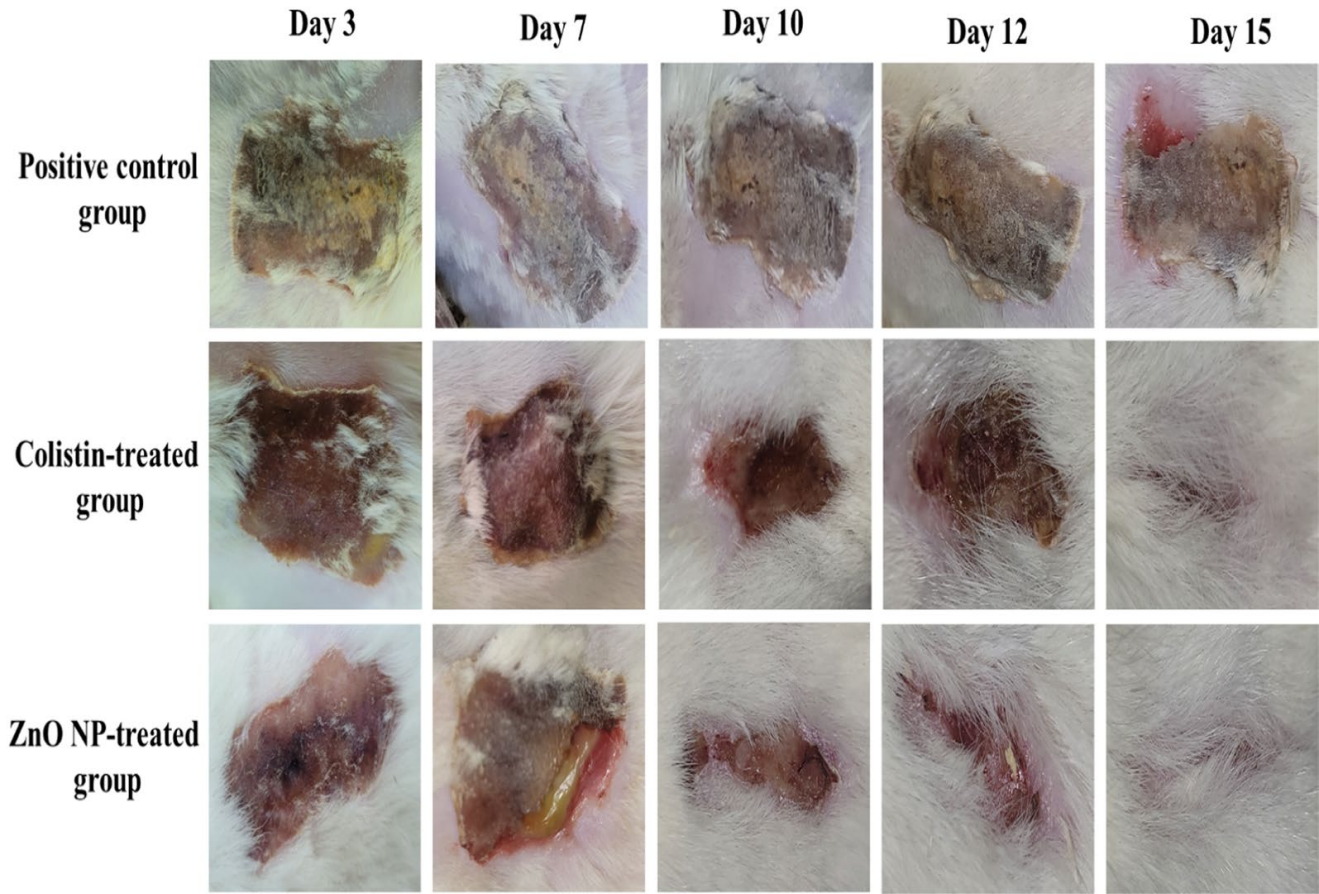
Clinical pictures of wounds of the positive control, colistin, and ZnO NP-treated groups

**Fig. 7:**
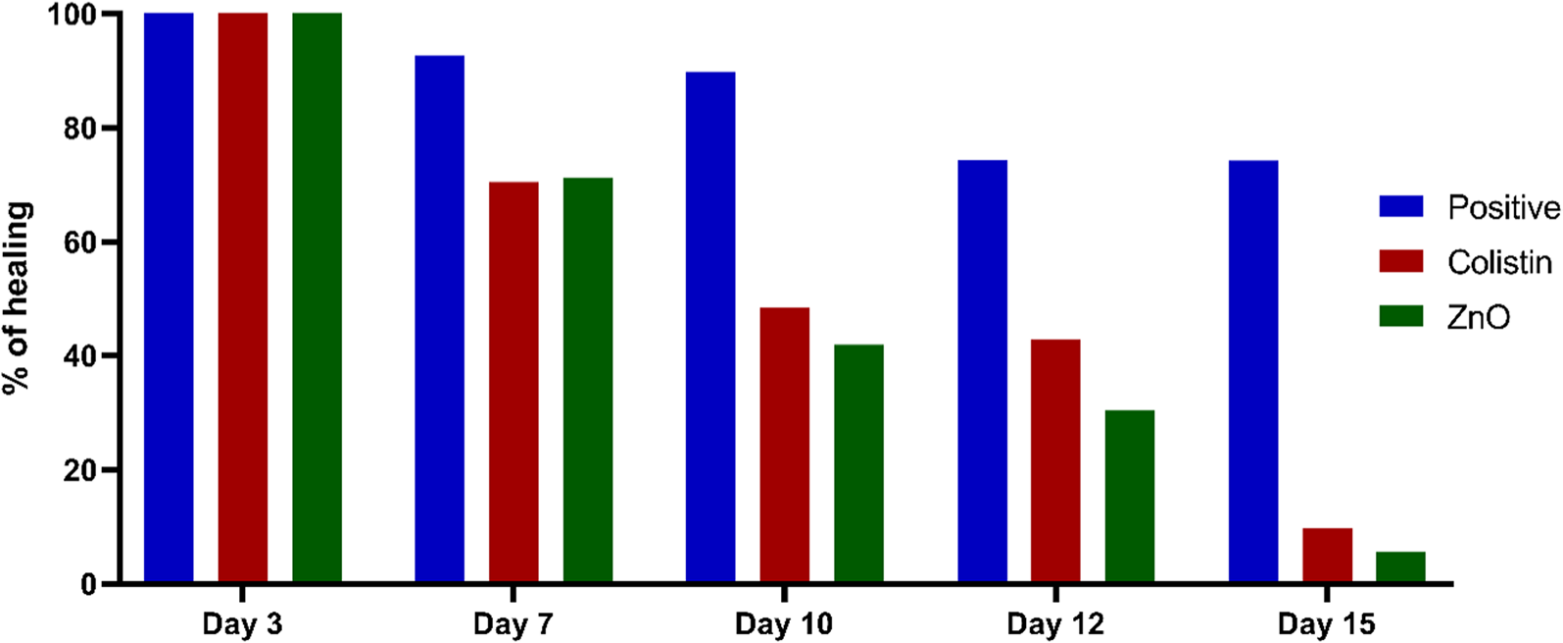
The mean ± SD of wound surface area percentage for the positive control, colistin-treated, and ZnO NP-treated groups

There was a brown wound with observed stiffness, erythema on day zero of treatment. On the third day of treatment, the wound began to be moist and covered by a scab with reddish-brown color in both colistin and ZnO NP-treated groups while in the positive control, it remained brown with presence of a thick, stiff and purulent scab. On the seventh day, the wound became moistier, less rounded, covered by a thin scab of more reddish color with less exudate in the colistin and ZnO NP-treated groups. On the contrary, the scale remained stiff with brown color in the positive control group. After 10 days, the wound became narrower in the ZnO NP-treated group with no sign of pus accumulation while it had an irregular round shape in the colistin-treated group and hair began to grow again in both groups. The positive control group did not show any sign of improvement. By the 12th day, the wound of the colistin and ZnO NP-treated groups became slightly narrower, and hair growth increased. Conversely, wounds of the positive control group kept their stiffness and their brown color. On the15^th^ day, wounds of both colistin and ZnO NP-treated groups were significantly minimized, leaving a small scar tissue that was covered by regrown hair. The scab of the positive control group finally peeled off showing the wound with presence of some pus underneath. Figure 6 demonstrates the wounds of the positive control, colistin, and ZnO NP-treated groups on days three, five, seven, 10, 12, and 15 of the experiment. Figure 7 represents the mean ± SD of the wound surface area percentage for the different experimental groups (Tables 3 and 4).

**Table 3:** Percentages of wound healing among the treated groups

Days	Positive	Colistin	ZnO NP
3	100	100	100
7	92.57596	70.42338	71.17111
10	89.88135	48.51925	41.92057
12	74.23486	42.85949	30.52911
15	74.16279	9.74553	5.62432

**Table 4:** The findings of the immunohistochemical staining

Group	IL-6 findings	TNF-α findings
Normal control	No to minimal cytoplasmic immunoreactivity in the fibrous tissue (A, a)	Minimal cytoplasmic immunoreactivity in a few fibroblasts (A, a)
Positive control	Intense cytoplasmic immunoreactivity in the fibrous tissue (B, b)	Moderate cytoplasmic immunoreactivity in the middle and surface of two-thirds of the fibrous tissue (B, b)
Colistin-treated group	Mild cytoplasmic immunoreactivity in the deeper parts of the fibrous tissue (C, c)	Mild cytoplasmic immunoreactivity in a few cells in the middle third of the fibrous tissue (C, c)
ZnO NP-treated group	Mild cytoplasmic immunoreactivity in the deeper half of the fibrous tissue (D, d)	Mild cytoplasmic immunoreactivity in a few scattered cells (D, d)

## Histopathological and immunohistochemical studies

**Fig. 8:**
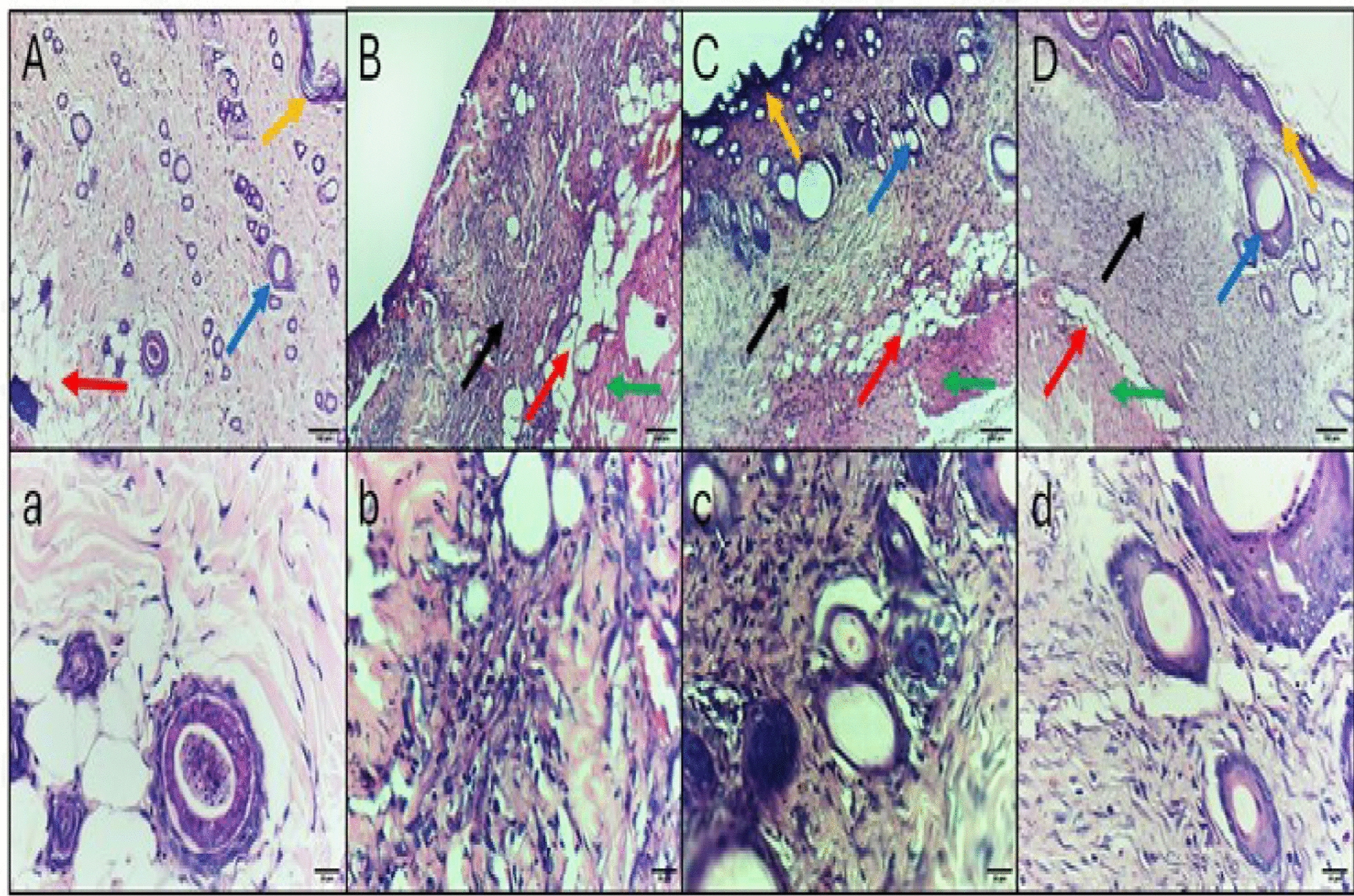
The H&E staining micrographs of the skin tissues of **A**, **a** Normal control displaying thin fibrous tissue, thin keratinized epithelium, and several hair follicles. **B**, **b** Positive control group displaying dense fibrosis, no epithelium or keratin formation, and no hair follicles. **C**, **c** Colistin-treated group displaying less organized fibrosis, primary surface epithelization with keratinization, and multiple hair follicles formation. **D**, **d** ZnO NP-treated group displaying less fibrosis, mature surface epithelization with keratinization, and few hair follicles formation. Black arrow: fibrosis (collagen bundles), red arrow: subcutaneous fat, orange arrow: keratinized epithelium, green arrow: muscle layer, blue arrow: hair follicles. Upper raw original magnification 10 ×, lower raw 40 × and scale bar 100 µm and 20 µm, respectively

**Fig. 9:**
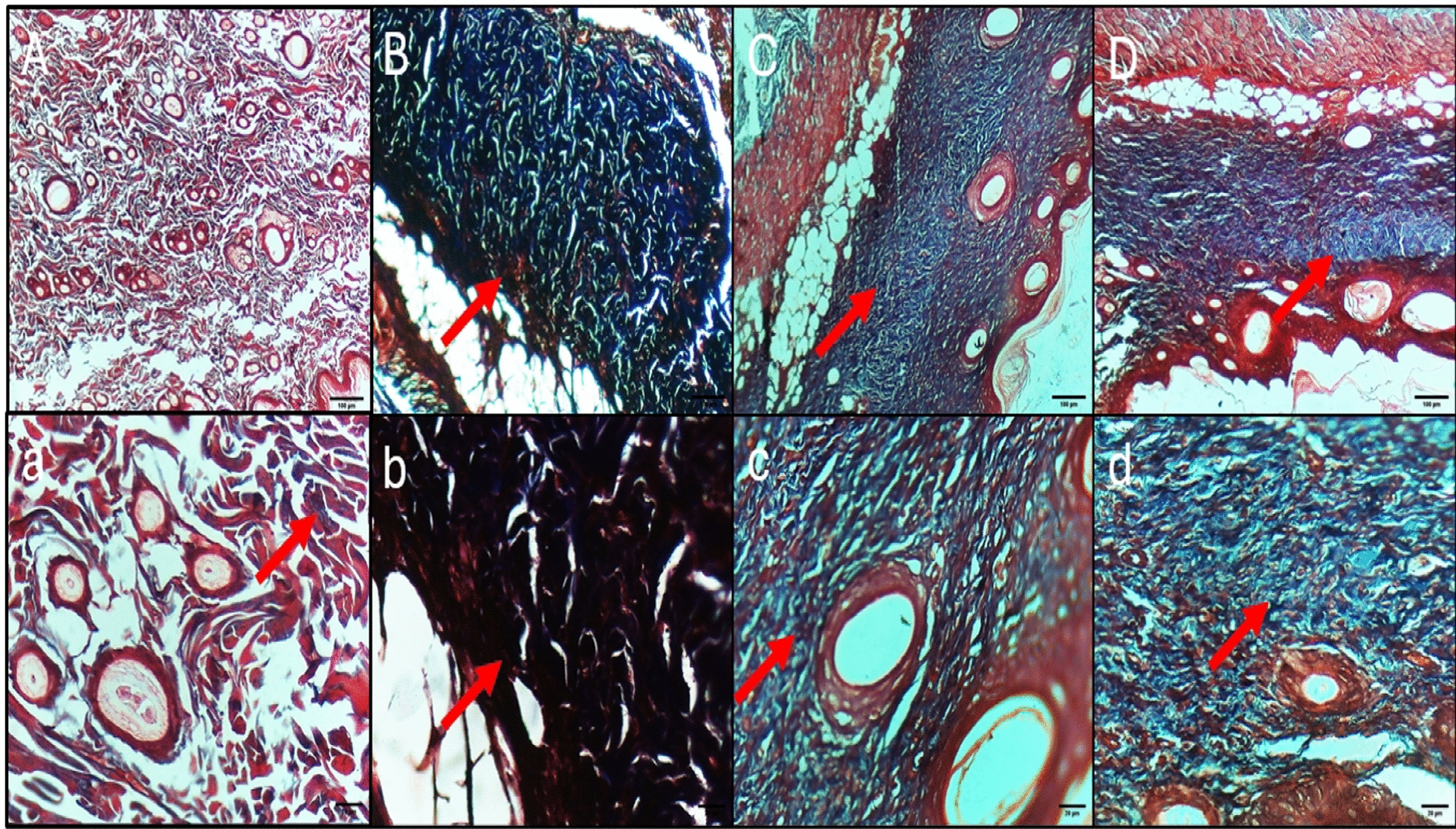
Masson’s trichrome staining micrographs of the skin tissues of **A**, **a** Normal control displaying thin fibrosis in the dermal layer. **B**, **b** Positive control group showing high fibrosis in the dermal layer. **C**, **c** Colistin-treated group displaying less fibrosis and epithelium formation. **D**, **d** ZnO NP-treated group displaying less fibrosis and epithelium formation. Upper raw original magnification 10 ×, lower raw 40 × and scale bar 100 µm and 20 µm, respectively

**Fig. 10:**
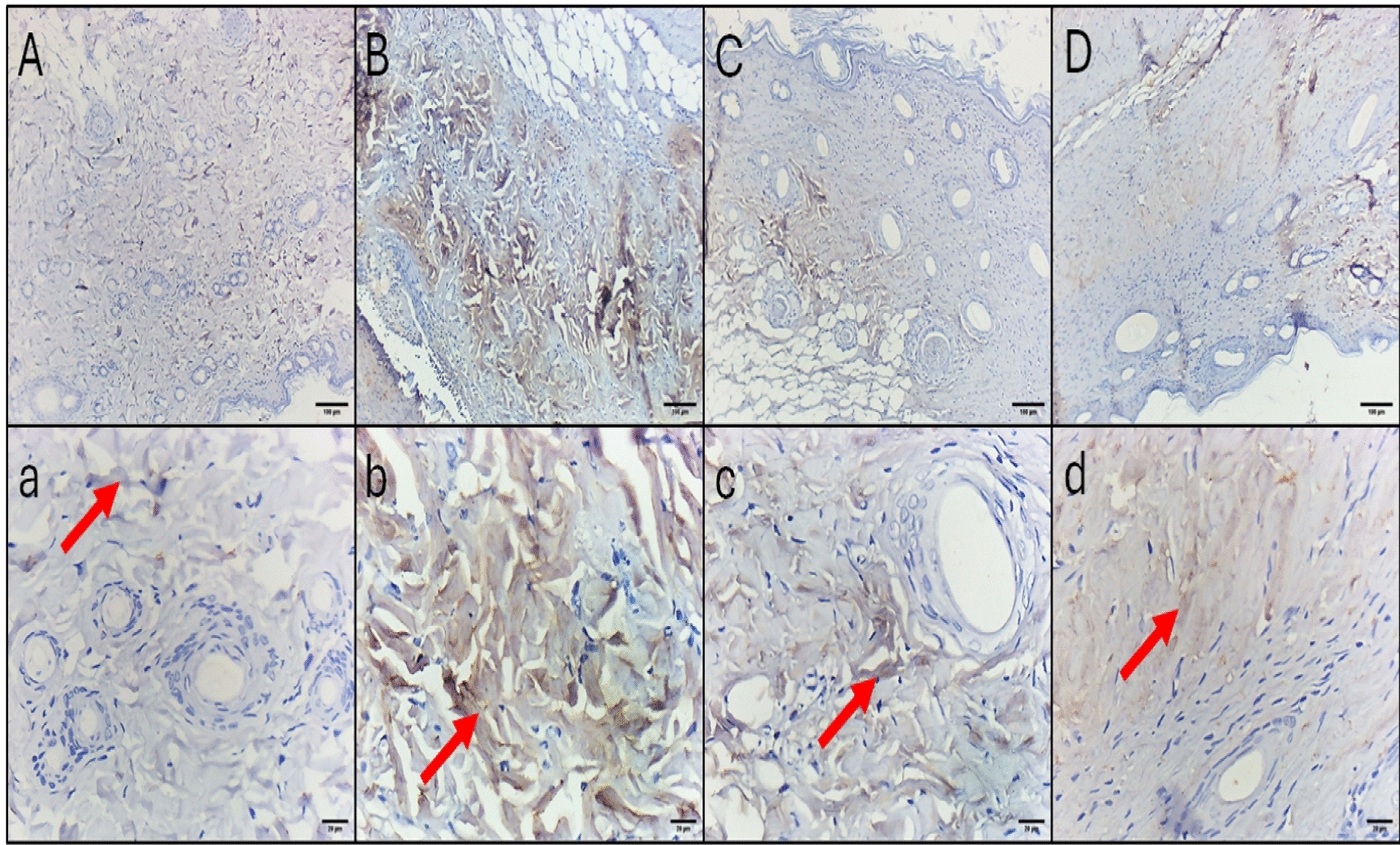
The IL-6 immunohistochemical staining micrographs of skin tissues of **A**, **a** Normal control displaying no to minimal cytoplasmic immunoreactivity in the fibrous tissue. **B**, **b** Positive control group displaying intense cytoplasmic immunoreactivity in the fibrous tissue. **C**, **c** Colistin-treated group displaying mild cytoplasmic immunoreactivity in the deeper parts of the fibrous tissue only. **D**, **d** ZnO NP-treated group displaying mild cytoplasmic immunoreactivity in the deeper half of the fibrous tissue. Upper raw original magnification 10 ×, lower raw 40 × and scale bar 100 µm and 20 µm, respectively

**Fig. 11:**
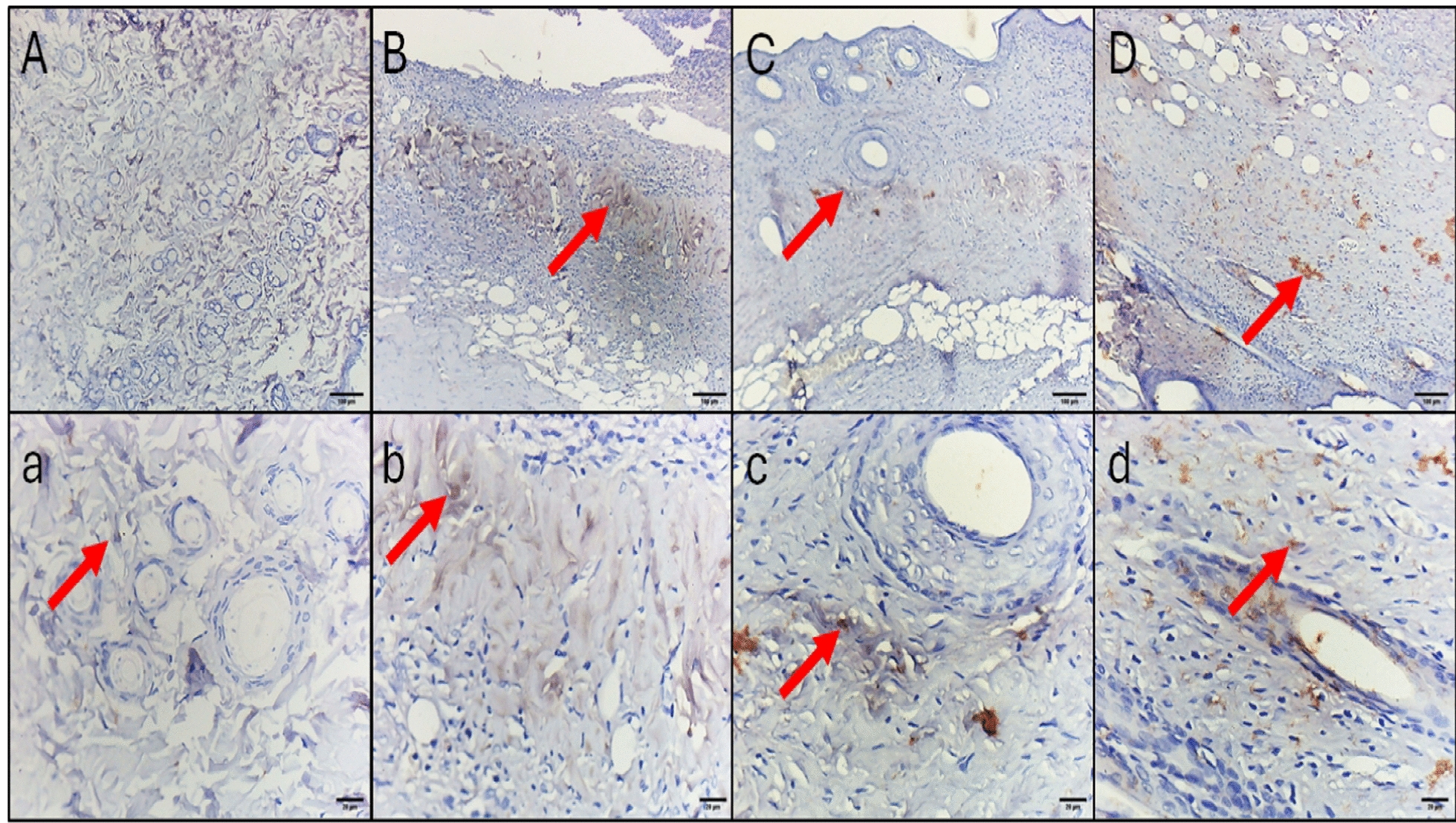
The TNF-α immunohistochemical staining micrographs of skin tissues of **A**, **a** Normal control displaying minimal cytoplasmic immunoreactivity in few fibroblasts. **B**, **b** The positive control group displayed moderate cytoplasmic immunoreactivity in the middle and surface of two-thirds of the fibrous tissue. **C**, **c** The colistin-treated group displayed mild cytoplasmic immunoreactivity in a few cells in the middle third of the fibrous tissue. **D**, **d** ZnO NP-treated group displaying mild cytoplasmic immunoreactivity in a few scattered cells. Upper raw original magnification 10 ×, lower raw 40 × and scale bar 100 µm and 20 µm, respectively

The microscopic histological features of the wounds are revealed in Figs. 8 and 9. The IL-6 and TNF-α immunohistochemical staining micrographs of the skin tissue sections are revealed in Figs. 10 and 11.

## Discussion

MDR bacteria represent a noteworthy threat worldwide as effective treatment options have become more limited. The alarming emergence of MDR among *A. baumannii* causes a high incidence of mortality and morbidity. Carbapenems are considered an important class of antibiotics for treating serious infections, but CRA has been spreading at a growing and alarming rate lately [37].

The findings of our study are worrying because all the tested 20 (100%) isolates were resistant to meropenem and imipenem. Previous literature reported that all 77 (100%) isolates resisted imipenem [38]. A study in the Kingdom of Saudi Arabia revealed that the spreading percentage of carbapenem-resistance among *A. baumannii* isolates against meropenem and imipenem was 84% and 81% of the strains, respectively [39]. Another study from Saudi Arabia revealed that 24 isolates (89%) of *A. baumannii* were resistant to one or more carbapenems (imipenem and meropenem) [40]. Another retrospective study from Saudi Arabia also revealed that almost all isolates of *A. baumannii* were carbapenem-resistant (98%) [41]. Recommendations for employing efficient infection control procedures and an appropriate antimicrobial stewardship program are compulsory to combat these isolates [42].

In our study, *bla*_KPC_ was the most predominant carbapenemase gene in 85% of the isolates after that *bla*_VIM_ gene which was revealed in 65% of the isolates. That encounter a previous study in which *bla*_IMP_ was among the most commonly identified carbapenemase-production genes with an incidence of 94% [43]. Another report that disagrees with our results detected *bla*_NDM_ at a rate of 3.7%, and *bla*_VIM_ and *bla*_IMP_ weren’t perceived in any isolate [44]. Our results agree with another study where *bla*_VIM_ was perceived in all 77 isolates, and the detection rate of blaIMP and blaNDM was 67.53% and 31.17%, respectively [38].

The need to discover and develop new drugs to overcome such MDR isolates has become an increasing demand globally. Still, unfortunately, the speed of drug development processes is not quick enough to cope with this demand. As a result, some non-antibiotic approaches are being investigated for their antimicrobial activity to develop new medications to combat such serious infections. Green ZnO NP has gained a recent concern as a new approach to antimicrobial agents because they are cheaper, more durable, and more stable than traditionally manufactured NPs [45]. ZnO NPs, either alone or combined with antibiotics, are reported to display multiple activities on MDR bacteria, adopting various action modes [46].

Nowadays, multiple researchers are utilizing a novel scope of NP biosynthesis via microorganisms, enzymes, and plants [47]. Cyanobacteria are documented to play a tremendous role in the biosynthesis of NP [48]. They are superior to plants as they don’t require different resources of land and water in addition to their ability to produce a huge biomass at a very low cost [49]. *A. maxima* a filamentous blue-green cyanobacteria that is categorized under microalgae are known for its nutritional value as it contains high protein and nutrients content [50]. The XRD peaks revealed the hexagonal wurtzite-type structure of ZnO NP comparable to previously published data [51, 52]. FTIR exhibited that the key bands of absorption were around 3700, 3000, 1700, 1500, 1200 cm^−1^ that match to O–H, C–H, CO_2_, asymmetric C=O, and symmetric C=O modes, respectively. The band around 400 is characteristic of the Zn–O bond and endorses the ZnO occurrence [34, 51]. SEM disclosed the excellent dispersion and the hexagonal shape of the biosynthesized ZnO NP, which agrees with the prior literature [53]. TEM also revealed that ZnO NP had a particle size of less than 100 nm, which agrees with Santhoshkumar et al*.* [54]. It is documented that the reduced particle size of the NP provides it with greater functionality as an antimicrobial owing to its larger surface-to-volume ratio [55].

The mechanism of the antibacterial activity of the ZnO NP relies on numerous factors, including their morphology, concentration, and composition [56]. Previous studies reported the antibacterial activity of ZnO NP synthesized using different algal extracts against different bacterial infections, such as *Staphylococcus aureus*, *Bacillus cereus*, *Salmonella enterica*, and *Klebsiella pneumoniae* standard isolates [57–61].

*Acinetobacter baumannii* infections of burn wounds could hinder the process of wound healing and progress into sepsis and, subsequently death [62]. Thus, it is critical to reveal novel approaches for the treatment of CRA isolates, especially in burn wounds. Here, the green ZnO NP proved to have a strong effect against burn wounds infected with CRA, and its effect was comparable to colistin. The H&E staining of the skin sections of the ZnO NP-treated group showed low fibrosis, mature surface epithelization with keratinization, and few hair follicles formation. IHC exposed only mild cytoplasmic immunoreactivity of IL-6 and TNF-α in the skin tissues of the ZnO NP-treated group. IL-6 and TNF-α are inflammatory markers frequently produced in response to tissue injury and bacterial infections [63–66]. In general, the effect of ZnO NP and colistin on infected burns in rats was closely similar. In a previous study, ZnO NP formulated in oleogel showed a significant decrease in the necrosis and burn wound area on the 10th day of treatment compared to the 1st day and improved the microcirculation [67]. Another study on the wound-healing properties of green ZnO NP in rats showed that it had more efficacy than ZnO NP synthesized chemically [34].

The findings of this study align with those of other previous studies that highlight the high prevalence of carbapenem resistance among *A. baumannii* isolates [38]. Our study shows a 100% resistance rate to both meropenem and imipenem, which is slightly higher than some other reports [39]. As for carbapenemase gene detection, our study found *bla*_KPC_ to be the most prevalent gene, which disagrees with some other studies that identified *bla*_IMP_ as more common [43]. However, the detection of *bla*_VIM_ is consistent with other research, though prevalence rates are different [38]. Regarding the antibacterial activity of ZnO NP, the study supports the previous findings that green ZnO NP is effective against various bacterial infections and promotes wound healing more than the chemically synthesized ZnO NP [34, 67]. The results support this claim as they are comparable to traditional antibiotics like colistin.

## Conclusion

ZnO NP were biosynthesized from *A. maxima,* characterized, and evaluated for their efficacy as antibacterial agents in vitro and in vivo. The current study proved the biosynthesized ZnO NP's activity to be an effective compound against CRA isolates. This is due to the revealed antibacterial properties of the biosynthesized ZnO NP both in vivo and in vitro against the tested CRA isolates. Moreover, we observed that ZnO NP significantly diminished the inflammation triggered by the induced infection. As a result, this eco-friendly ZnO NP could be considered in future studies as an important, safe, and biocompatible treatment option to deal with such serious infections caused by MDR bacteria that pose a risk to public health.

## Supplementary Information

Supplementary Material 1.

## Data Availability

No datasets were generated or analysed during the current study.
