# Supplementary material for: Antibacterial and wound healing potential of biosynthesized zinc oxide nanoparticles against carbapenem-resistant Acinetobacter baumannii: an in vitro and in vivo study
Source: Microb Cell Fact. 2024 Oct 16;23:281. doi: 10.1186/s12934-024-02538-3 (PMC11484456; doi:10.1186/s12934-024-02538-3)
Supplement: Supplementary file 1 — Supplementary Material 1. [file 12934_2024_2538_MOESM1_ESM.docx]

**Table S1.** Sample types of collected *A. baumannii* isolates.

| **A. sample number** | **Sample type** |  |
| --- | --- | --- |
|  |  |  |
|  |  |  |
| **A1** | Wound |  |
| **A2** | Blood |  |
| **A3** | Wound |  |
| **A4** | Wound |  |
| **A5** | Pus |  |
| **A6** | Blood |  |
| **A7** | Blood |  |
| **A8** | Wound |  |
| **A9** | Blood |  |
| **A10** | Sputum |  |
| **A11** | Pus |  |
| **A12** | Urine |  |
| **A13** | Wound |  |
| **A14** | Blood |  |
| **A15** | Blood |  |
| **A16** | Blood |  |
| **A17** | Blood |  |
| **A18** | Sputum |  |
| **A19** | Urine |  |
| **A20** | Wound |  |

**Table S2.** Primer sequence for the PCR studies

| Amplicon Size (bp) | Sequence | Type | Gene's name |
| --- | --- | --- | --- |
| 780 | 5`… ATTGGTCTATTTGACCGCGTC | F | *bla*_VIM_ |
|  | 5`… TGCTACTCAACGACTGAGCG | R |  |
| 770 | 5`…ATGCGTGTATTAGCCTTATCGGC | F | *bla*_OXa-48_ |
|  | 5`… ACTTCTTTTGTGATGGCTTGGCGCA | R |  |
| 621 | 5`… GGTTTGGCGATCTGGTTTTC | F | *bla*_NDM-1_ |
|  | 5`… CGGAATGGCTCATCACGATC | R |  |
| 488 | 5`… CATGGTTTGGTGGTTCTTGT | F | *bla*_IMP_ |
|  | 5`… ATAATTTGGCGGACTTTGGC | R |  |
| 390 | 5`… CGTTGACGCCCAATCC | F | *bla*_KPC_ |
|  | 5`… ACCGCTGGCAGCTGG | R |  |

**Table S3.** Distribution of carbapenem resistance genes in tested isolates.

| **Isolate** | **VIM** | **KPC** | **NDM-1** | **IMP** | **OXA-48** |
| --- | --- | --- | --- | --- | --- |
| **A1** | (-) | (+) | (-) | (-) | (-) |
| **A2** | (-) | (+) | (-) | (+) | (-) |
| **A3** | (-) | (+) | (-) | (-) | (-) |
| **A4** | (-) | (+) | (-) | (-) | (-) |
| **A5** | (-) | (+) | (-) | (-) | (-) |
| **A6** | (-) | (+) | (-) | (-) | (-) |
| **A7** | (+) | (-) | (-) | (-) | (-) |
| **A8** | (-) | (+) | (-) | (-) | (-) |
| **A9** | (+) | (+) | (+) | (-) | (-) |
| **A10** | (+) | (+) | (+) | (-) | (+) |
| **A11** | (+) | (+) | (+) | (-) | (+) |
| **A12** | (+) | (+) | (+) | (+) | (-) |
| **A13** | (+) | (+) | (+) | (-) | (-) |
| **A14** | (+) | (+) | (+) | (-) | (+) |
| **A15** | (+) | (+) | (+) | (-) | (+) |
| **A16** | (+) | (+) | (+) | (+) | (+) |
| **A17** | (+) | (+) | (+) | (-) | (+) |
| **A18** | (+) | (-) | (+) | (-) | (+) |
| **A19** | (+) | (+) | (+) | (-) | (+) |
| **A20** | (+) | (-) | (-) | (-) | (-) |


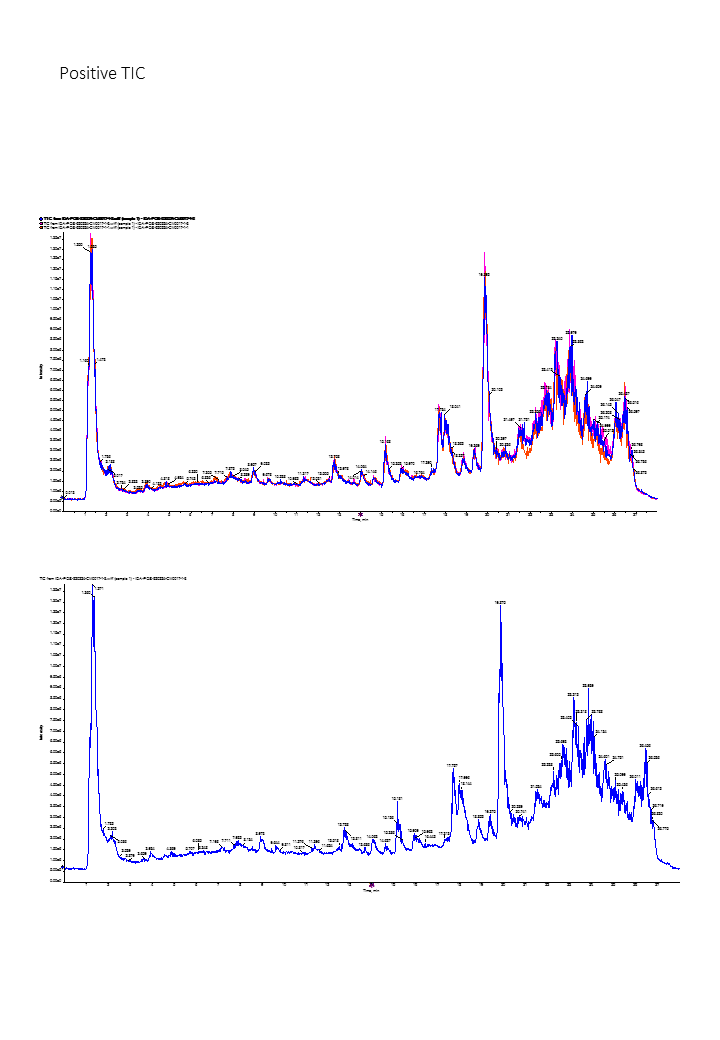


**Figure S1.** The negative mode total ion chromatograms (TIC) of *A. maxima*.
